# Dengue viruses in Papua New Guinea: evidence of endemicity and phylogenetic variation, including the evolution of new genetic lineages

**DOI:** 10.1038/emi.2017.103

**Published:** 2017-12-20

**Authors:** Peter R Moore, Andrew F van den Hurk, John S Mackenzie, Alyssa T Pyke

**Affiliations:** 1 Public Health Virology Laboratory, Forensic and Scientific Services, Coopers Plains, Queensland 4108, Australia; 2 Faculty of Medical Sciences, Curtin University, Perth, Western Australia 6102, Australia; 3 Division of Microbiology and Infectious Diseases, PathWest, Nedlands, Western Australia 6909, Australia

**Keywords:** dengue, dengue virus, Papua New Guinea, arbovirus, virus evolution, phylogenetics, endemic lineage

## Abstract

Dengue is the most common cause of mosquito-borne viral disease in humans, and is endemic in more than 100 tropical and subtropical countries. Periodic outbreaks of dengue have been reported in Papua New Guinea (PNG), but there is only limited knowledge of its endemicity and disease burden. To help elucidate the status of the dengue viruses (DENVs) in PNG, we performed envelope (E) gene sequencing of DENV serotypes 1–4 (DENV 1–4) obtained from infected patients who traveled to Australia or from patients diagnosed during local DENV transmission events between 2001 and 2016. Phylogenetic analysis and comparison with globally available DENV sequences revealed new endemic PNG lineages for DENV 1–3 which have emerged within the last decade. We also identified another possible PNG lineage for DENV-4 from 2016. The DENV-1 and 3 PNG lineages were most closely related to recent lineages circulating on Pacific island nations while the DENV-2 lineage and putative DENV-4 PNG lineage were most similar to Indonesian sequences. This study has demonstrated for the first time the co-circulation of DENV 1–4 strains in PNG and provided molecular evidence of endemic DENV transmission. Our results provide an important platform for improved surveillance and monitoring of DENVs in PNG and broaden the global understanding of DENV genetic diversity.

## INTRODUCTION

Responsible for significant socioeconomic and disease burdens within the tropics and sub-tropics, the recent and rapid geographical expansion of the dengue viruses (DENVs) has placed more than half of the world’s population at risk and resulted in a global infection rate of ~390 million per year.^1^ In the absence of a safe and effective vaccine for all four DENV serotypes, dengue remains the leading mosquito-borne viral disease of humans and nearly 75% of the population that are affected globally reside in the Asia-Pacific region.^1, 2^

Belonging to the genus *Flavivirus*, family *Flaviviridae*, the DENVs are single stranded, positive-sense RNA viruses comprised of four antigenically distinct serotypes (DENV 1–4).^3^ Partial or complete nucleotide sequencing of the envelope (E) gene and analysis of strain variation has further classified DENVs within each serotype into genetically distinct genotypes and lineages.^4, 5, 6, 7^ Separate, basal positioning of sylvatic DENV strains within phylogenetic trees also suggests each DENV serotype has evolved independently from sylvatic DENV progenitors involving non-human primate hosts.^4, 7, 8^

DENVs were identified as a major cause of morbidity amongst soldiers deployed to Asia and the Pacific during World War II. The first recovered strains of DENV were isolated in 1944 and included DENV-1 (US/Hawaii) and DENV-2 (PNG/New Guinea C) strains. DENV-3 (H-87) and DENV-4 (H-241) viruses were first isolated a few years later in the 1950s during dengue epidemic activity in the Philippines.^9, 10, 11^ The major demographic and societal changes that have occurred since World War II leading to massive unplanned urbanization, the increased movement of viruses in infected humans through modern transportation, and the lack of effective mosquito control, have all contributed to the profound global geographic expansion of the DENVs and their anthropophilic *Aedes* mosquito vectors.^8, 12, 13, 14, 15, 16^

Over 100 countries are currently burdened with endemic DENV transmission and at least 31 countries and territories in the Western Pacific have reported dengue cases in the last two decades.^17^ Situated in the southwestern Pacific, Papua New Guinea (PNG) is located in close geographical proximity to several DENV affected countries within the Pacific and Southeast Asia, and Western and Sandaun Provinces of PNG share a land border with Papua Province of Indonesia. Despite this, only sporadic DENV activity within PNG is reported and the dynamics of transmission is unclear.

The first recorded epidemic of dengue in PNG was a DENV-2 outbreak which occurred in Rabaul in 1971 and resulted in over 1100 cases.^18^ Additional DENV outbreaks from undetermined DENV serotype(s) were also reported in 1976 and 1983.^19^ In a serological study conducted in the Madang Province on the northern coast of PNG, between October 2007 and June 2008, acute dengue infections were identified in 8% (46/578) of febrile outpatients.^20^ In 2015–2016, DENV outbreaks were reported in Daru, Western Province, and in Port Moresby resulting in 170 and 15 cases, respectively.^21^

The role of Pacific island nations as epicentres for mosquito-borne viral transmission and disease has been highlighted by recent DENV, CHIKV and ZIKV epidemics.^22, 23, 24, 25, 26, 27, 28^ The continued transmission of DENV in PNG coupled with an escalation in air travel has directly impacted on neighboring countries harboring *Ae. aegypti* and *Ae. albopictus* mosquitoes. Although DENVs are not endemic in Australia, several DENV outbreaks in northern Queensland have been initiated by viremic travelers from PNG, including DENV-2 outbreaks in 1996,^29^ 2003/2004^30^ and 2016. This has necessitated costly and rigorous disease surveillance and mosquito vector control measures to minimize local DENV transmission.^29, 30, 31, 32, 33^

As part of our ongoing surveillance and monitoring of imported DENVs, we performed E gene phylogenetic analysis of DENVs obtained from travelers to Australia or from patients infected during local outbreaks in north Queensland, Australia between 2001 and 2016. In the current study, we provide the first molecular evidence for the co-circulation of DENV 1–4 in PNG and have identified the presence of endemicity, including the evolution of new genetic lineages. Our data highlight the continual introduction of genetically diverse DENV strains into PNG and the increased diversity of PNG strains which are subsequently introduced into Australia.

## MATERIALS AND METHODS

### Ethics statement

All methods were performed in accordance with approved ethical guidelines and all patient samples were anonymized. Ethical approval for this study and the use of RNA extracted from existing patient samples held in Public Health Virology was granted by the Forensic and Scientific Services Human Ethics Committee.

### RNA extraction and nucleotide sequencing

To minimize potential nucleotide changes induced by downstream cell culture virus isolation techniques, viral RNA extraction, reverse transcription polymerase chain reactions (RT-PCRs) and nucleotide sequencing were primarily performed directly from patient serum samples as previously described.^32, 34^ Briefly, viral RNA was extracted from 200 μL of patient serum using the BioRobot Universal System and QIAamp One-For-All Nucleic Acid Kit (Qiagen, Australia), or EZ1 and Virus Mini Kit v2.0 (Qiagen, Australia), or manually from 140 μL of isolate culture supernatant using the QIAamp viral RNA extraction kit (Qiagen, Australia). Amplification was performed using 5 μL of RNA, specific RT-PCR DENV 1–4 primers^32^ and the Superscript III/Platinum Taq High Fidelity One-Step RT-PCR System (Invitrogen, USA). Nucleotide sequencing of complete DENV E genes (1485 nt for DENV-1, DENV-2 and DENV-4 and 1479 nt for DENV-3) was performed using the Big Dye Terminator version 3.1 cycle sequencing kit (Life Technologies, USA) and specific primers.^32^

**Figure 1:**
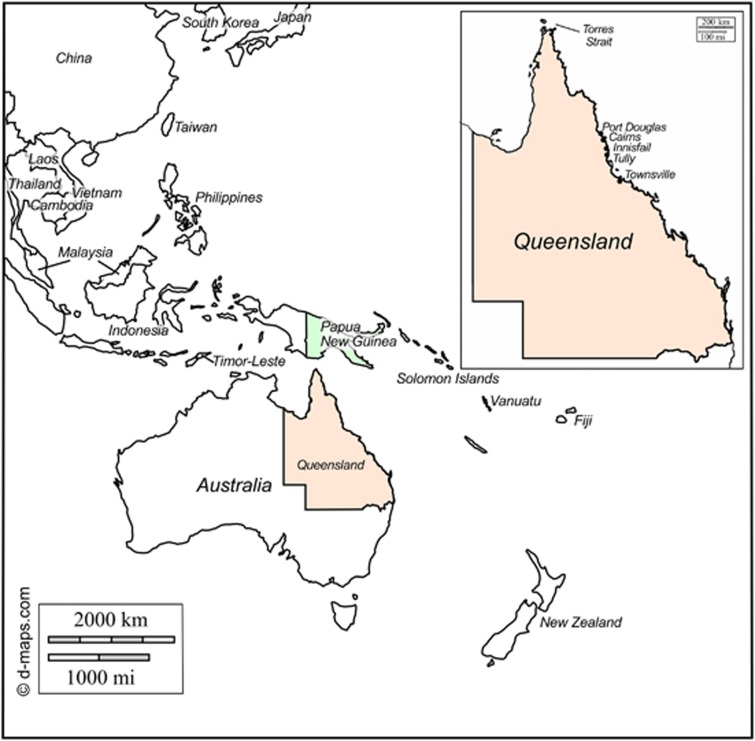
Map of Australia, PNG and surrounding regions, including inset map of Queensland, Australia showing locations of local outbreaks resulting from imported PNG DENV strains. Map was adapted from a template obtained from d-maps.com (http://d-maps.com/carte.php?num_car=3262&lang=en).

We determined 60 DENV-1, 59 DENV-2, 57 DENV-3 and 20 DENV-4 new E gene nucleotide sequences from viremic patients traveling to Australia or from patients diagnosed during local DENV transmission in north Queensland, Australia between 2001 and 2016 (Supplementary Table S1). These included 12 DENV-1, 11 DENV-2, 11 DENV-3 and 2 DENV-4 sequences from patients who had traveled from PNG. A map of Queensland, Australia, PNG and surrounding regions is shown in Figure 1. Specific residential location and demographics within PNG was not available for the patients.

### Phylogenetic analysis

To determine the genetic and evolutionary relationships between circulating PNG DENV strains and their relatedness to other strains found worldwide, we compared our DENV E gene sequences with globally available DENV 1–4 sequences from GenBank, excluding sylvatic, and recombinant and chimeric DENV viral sequences which contained two or more parental sequences. For each of the DENV serotypes, this resulted in very large sequence data sets of the following sizes and included DENV-1, 4467 sequences, DENV-2, 3369 sequences, DENV-3, 2046 sequences and DENV-4, 1368 sequences. Due to the extremely large number of sequences involved for each serotype, multiple sequence alignments were performed using the Multiple Alignment using Fast Fourier Transform (MAFFT) program version 7.222 and Geneious version 10.0.9 software.^35, 36, 37^ Approximately maximum-likelihood phylogenetic trees were inferred for each DENV serotype using FastTree version 2.1.5 software and the generalized time-reversible (GTR) substitution model of nucleotide evolution with the default setting of 20 for rate categories of sites.^38^ For each tree, the reliability of each split was estimated and validated using Shimodaira-Hasegawa-like local support values^38^ with 1000 resamples. All phylogenetic trees were graphically viewed in FigTree version 1.4.3 (http://tree.bio.ed.ac.uk/software/figtree/).

## RESULTS

In order to demonstrate the phylogenetic relatedness of available DENV strains from PNG and to assess the development of established lineages, we analyzed globally available DENV 1–4 E gene sequences in comparison with new sequences from 196 strains, which were imported into Australia by travelers, or were derived from local outbreaks in Queensland, Australia between 2001 and 2016. A summary of the co-circulating PNG DENV 1–4 strains and their respective genotype groups are provided in Table 1.

### Prevalence of DENV 1–4 genotypes in PNG sequences

We investigated the genotypic distribution of DENV 1–4 serotypes in PNG from 2001 to 2016 using phylogenetic analysis of 63 nucleotide sequences obtained from viremic patients who traveled to Australia and two additional PNG DENV-2 sequences from 2008 to 2013 (GenBank accession numbers FJ906959 and KU517845, respectively). The following distribution of genotypes within each DENV 1–4 serotype was obtained: DENV-1 (19 sequences), 5 (26%) genotype I, 14 (74%) genotype IV; DENV-2 (27 sequences), 1 (4%) Asian genotype II, 26 (96%) Cosmopolitan genotype; DENV-3 (17 sequences), 1 (6%) genotype III, 16 (94%) genotype I; DENV-4 (2 sequences), 2 (100%) genotype II.

Previous studies of DENV 1–4 serotypes have demonstrated multiple lineages that are often geographically based.^7^ The predominant genotypes for each of the DENV 1–4 serotypes (DENV-1/genotype IV, DENV-2/Cosmopolitan genotype, DENV-3/genotype I and DENV-4/genotype II) corresponded to previously reported genotypes which are highly prevalent in the Australasian/Pacific regions.^4, 5, 6, 7, 39, 40, 41^ The two genotype II DENV-4 sequences represent the first reported DENV-4 strains from PNG.

### Phylogenetic analysis

**Figure 2:**
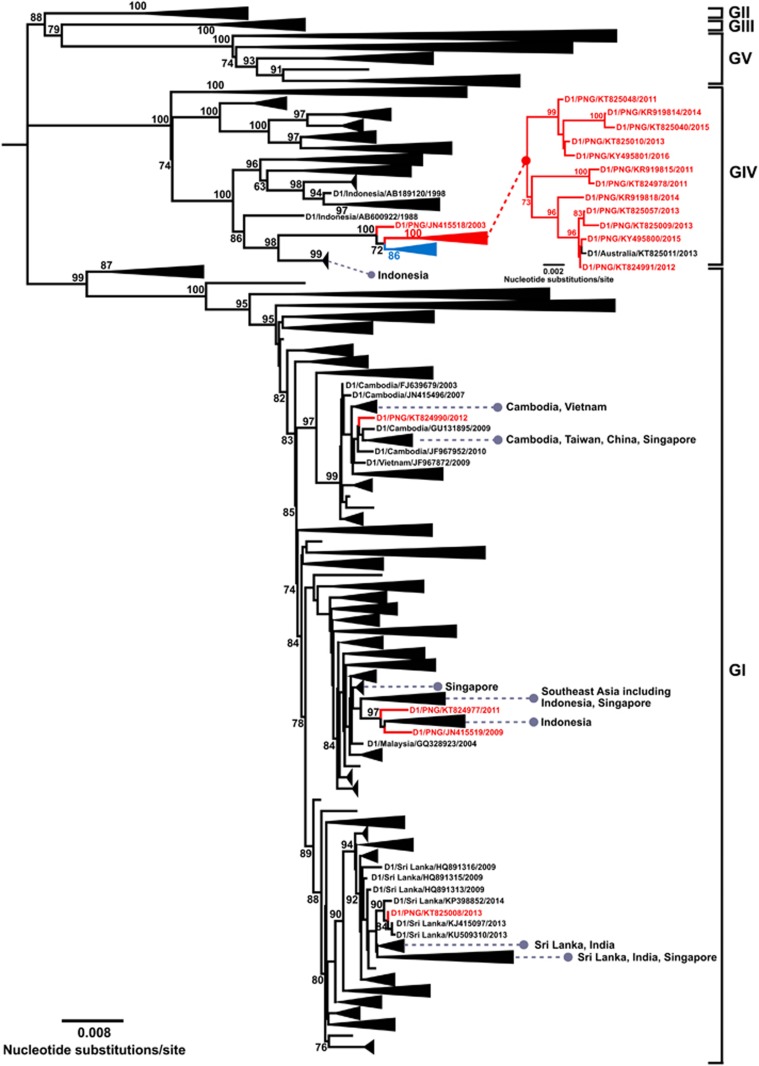
Maximum-likelihood phylogenetic tree of 4467 DENV-1 envelope (E) gene sequences (1485 nucleotides). The midpoint rooted phylogenetic tree, which includes 12 new sequences from viremic travelers returning from PNG to Australia, was inferred using FastTree and the generalized time-reversible (GTR) nucleotide substitution model.^38^ Percentage Shimodaira-Hasegawa-like local support values are shown for key nodes estimated from 1000 resamples. To emphasize positions of sequences from PNG and accommodate the very large sample size, PNG sequences are colored red among collapsed phylogenetic clusters and geographical locations of closely related sequences are shown. The inset phylogenetic tree within genotype IV depicts the established, endemic PNG lineage. A closely related lineage containing DENV-1 2002–2003 sequences from the Pacific is shown colored blue. Horizontal branch lengths are proportional to the bar representing the number of nucleotide substitutions/site.

**Figure 3:**
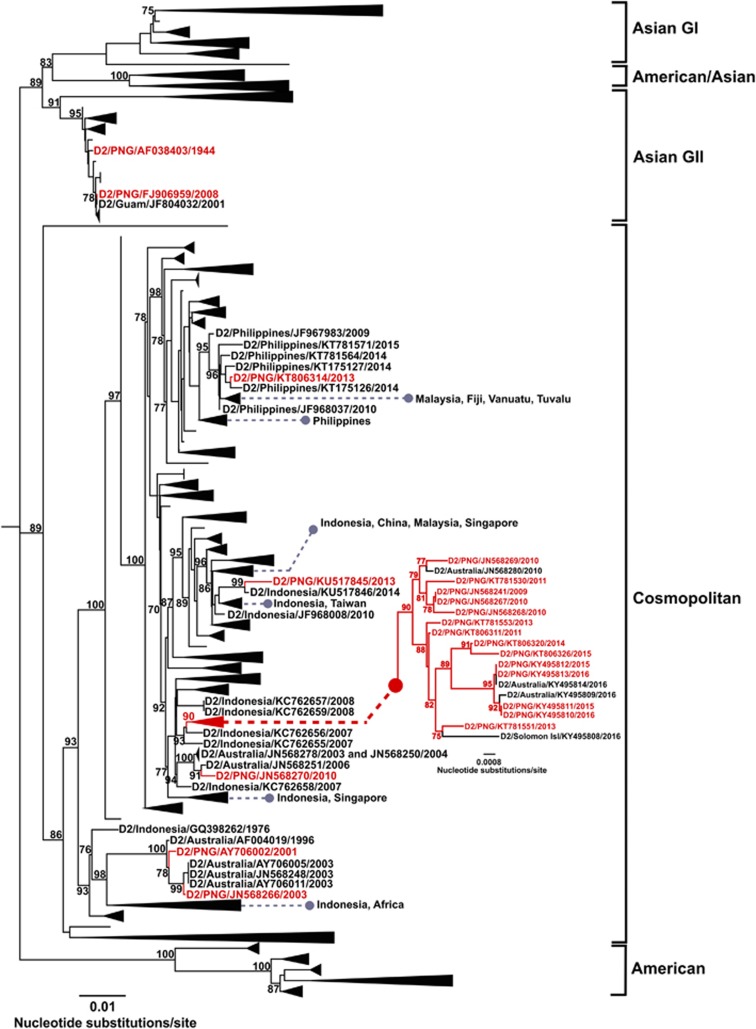
Maximum-likelihood phylogenetic tree of 3369 DENV-2 envelope (E) gene sequences (1485 nucleotides). Details of the phylogenetic tree analysis and graphical features are as provided in Figure 2 legend. The phylogenetic tree includes 11 new PNG sequences. The inset phylogenetic tree represents the established, endemic PNG DENV-2 lineage within the Cosmopolitan genotype.

**Figure 4:**
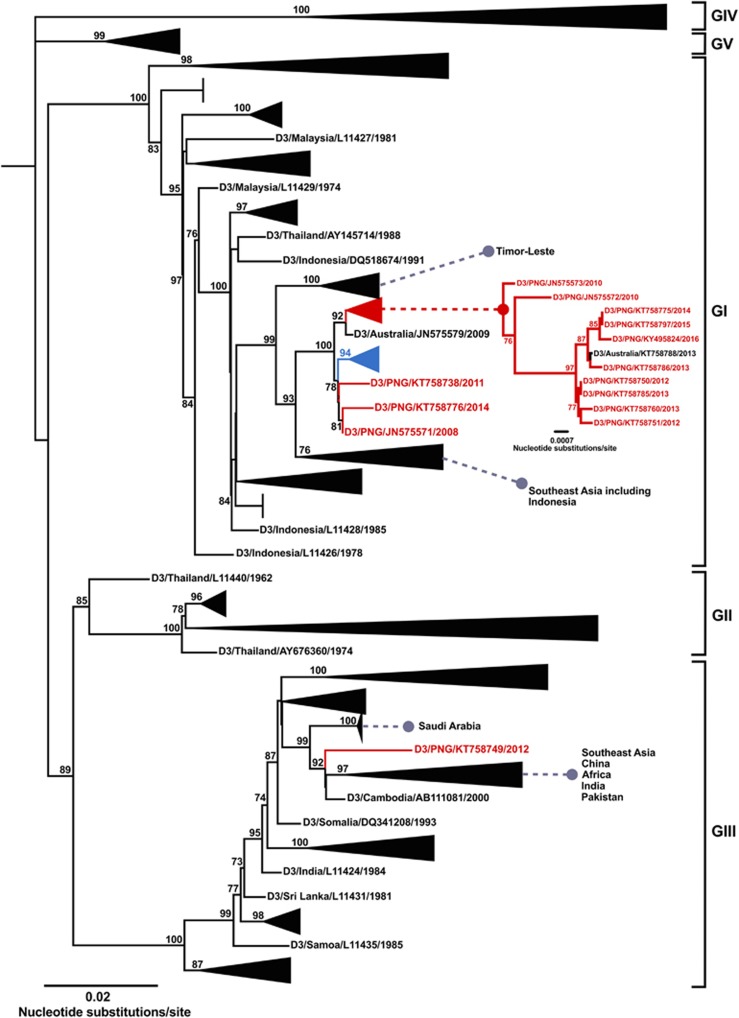
Maximum-likelihood phylogenetic tree of 2046 DENV-3 envelope (E) gene sequences (1479 nucleotides). Details of the phylogenetic tree analysis and graphical features are as given for Figure 2 legend. The phylogenetic tree includes 11 new PNG sequences. The established, endemic DENV-3 PNG lineage within genotype I is shown in the inset phylogenetic tree and another Pacific lineage containing sequences sampled between 2012 and 2016 is colored blue.

Overall, our phylogenetic analysis of DENV 1–3 demonstrated that most of the PNG sequences we analyzed grouped together within their respective serotypes and that each group stemmed from nodes demonstrating high-local support values. The persistent circulation of each of the DENV 1–3 PNG groupings over several years also indicated endemic transmission, affording the discovery of new and established lineages within PNG. Consistent with the above findings which demonstrated the prevalent DENV 1–4 genotypes among sequences obtained from patients in Australia, the genotypic placements of these lineages were DENV-1/genotype IV, DENV-2/Cosmopolitan genotype and DENV-3/genotype I, respectively (Figures 2, 3, 4). The PNG DENV 1–3 lineages had emerged and co-circulated within the last decade and contained sequences from locally acquired DENV cases from Australia. The DENV-2 PNG lineage also contained a 2016 sequence from the Solomon Islands (GenBank accession number KY495808). The remaining PNG DENV 1–3 sequences which did not group in the distinct PNG lineages demonstrated notable genetic diversity and were most were closely related to sequences from Indonesia or elsewhere in Southeast Asia or to sequences from Australia and the Pacific. Detailed phylogenetic findings for each DENV serotype are provided below.

### DENV-1

We analyzed 4467 DENV-1 E gene sequences, including 60 new sequences from viremic travelers (12 from PNG) or from Australian patients with locally acquired DENV infections. Of the new and globally available PNG sequences, 12 sequences (sampled between 2011 and 2016), grouped in a distinct lineage within genotype IV, together with a 2013 sequence from Port Douglas, north Queensland, Australia (GenBank accession number KT825011). Two major clades within the lineage were also evident depicting the diversity of co-circulating DENV-1 strains.

The PNG lineage was most closely related to a lineage from the Pacific containing 2002–2003 sequences from the Solomon Islands, Fiji, Yap Island, Marshall Islands and Australia (Figure 2, lineage colored blue).^27^ This Pacific lineage was distinct from other Pacific DENV-1 strains responsible for outbreaks in Palau, Samoa, New Caledonia and Tahiti between 2000 and 2003, suggesting these viruses were introduced into the Pacific from different locations in Southeast Asia.^27^

Both the PNG and Pacific lineages were most closely related to a PNG 2003 sequence (GenBank accession number JN415518). In turn, these sequences were most closely related to 1988 sequences from Indonesia (GenBank accession numbers AB074761, AB600923 and AB600924). These phylogenetic findings highlight several potential transmission routes including possible introduction of DENV-1 from Indonesia into PNG before further spread to Australia. Similarly, the Pacific lineage may has been derived from the PNG lineage or related viruses introduced from Indonesia.

Four additional genetically diverse PNG DENV-1 sequences were also demonstrated belonging to genotype I (GenBank accession numbers KT824990, KT824977, JN415519 and KT825008). These DENV-1 PNG sequences were closely related to sequences from Southeast Asian countries including Cambodia, Indonesia and Sri Lanka.

### DENV-2

A total of 3369 DENV-2 E gene sequences were analyzed which incorporated 59 new sequences (11 from PNG). Fourteen of the available PNG sequences (sampled between 2009 and 2016) fell into a distinct lineage within the Cosmopolitan genotype, which also included three Australian sequences (GenBank accession numbers JN568280, KY495814 and KY495809) and one sequence from the Solomon Islands (GenBank accession number KY495808) obtained in 2016. This lineage was most closely related to 2007 sequences from Indonesia (GenBank accession numbers KC762656 and KC762655). Similar to the PNG DENV-1 lineage, the DENV-2 PNG lineage also demonstrated genetic diversity within the group with the largest clade containing four PNG sequences (GenBank accession numbers KY495810–KY495813), and two Australian sequences (GenBank accession numbers KY495809 and KY495814) obtained from 2015 to 2016. This reflects the increased DENV activity in 2015–2016 due to recently reported PNG outbreaks ^21^ and further spread to Australia in 2016.^42^

Five other genetically diverse PNG sequences also belonging to the Cosmopolitan genotype were identified. The PNG DENV-2 sequences (GenBank accession numbers KT806314, KU517845 and JN568270) were most closely related to sequences from the Philippines, Indonesia and Australia, respectively. Two other PNG sequences (GenBank accession numbers AY706002 and JN568266) were also related to DENV-2 strains locally transmitted in Australia in 2003 and are highly suggestive of PNG being the source of these Australian outbreak strains. Interestingly, another Australian DENV-2 sequence from the Torres Strait, 1996 (GenBank accession number AF004019) is positioned basally in relation to AY706002 from 2001 to the 2003 Australian and JN568266 sequences. The 1996–1997 DENV-2 Torres Strait outbreak only lasted 28 weeks and the virus did not become established.^29^ The closest lineage to this grouping contains two sequences from Indonesia, 1976 (GenBank accession numbers GQ398260 and GQ398261) and an African lineage circulating since 1983. This suggests that the unidentified ancestral lineage of the DENV-2 PNG 2001 strain had existed in PNG prior to 1996 and was then introduced into the Torres Strait, or had been introduced from elsewhere, such as Indonesia. No further evidence exists of this group or its continued transmission. The remaining two PNG sequences analyzed belong to Asian genotype II and include the prototype 1944 New Guinea C strain and a PNG 2008 sequence (GenBank accession numbers AF038403 and FJ906959, respectively).

### DENV-3

Our DENV-3 phylogenetic data set was comprised of 2046 E gene sequences which included 57 new sequences, (11 of which were from PNG). Ten PNG sequences formed an established lineage group within genotype I, together with one 2013 sequence from Port Douglas, Australia (GenBank accession number KT758788). Within the PNG lineage, basally positioned PNG 2010 sequences (GenBank accession numbers JN575573 and JN575572) were genetically distinct in comparison with a later emerging clade containing 8 PNG sequences and KT758788 from the period 2012 to 2014. The PNG DENV-3 lineage was most closely related to the Townsville, Australia, 2009 sequence (GenBank accession number JN575579). These sequences were then phylogenetically related to a Pacific clade containing sequences from Fiji, Vanuatu, Samoa, Tonga, Nauru Island and the Solomon Islands (sampled between 2012 and 2016), and three additional PNG sequences (GenBank accession numbers KT758738, KT758776 and JN575571) sampled from the years 2011, 2014 and 2008, respectively. Basal to this group were lineages containing Indonesian and other Southeast Asian sequences. One additional PNG 2012 sequence (GenBank accession number KT758749), which was quite genetically diverse belonged to genotype III.

### DENV-4

**Figure 5:**
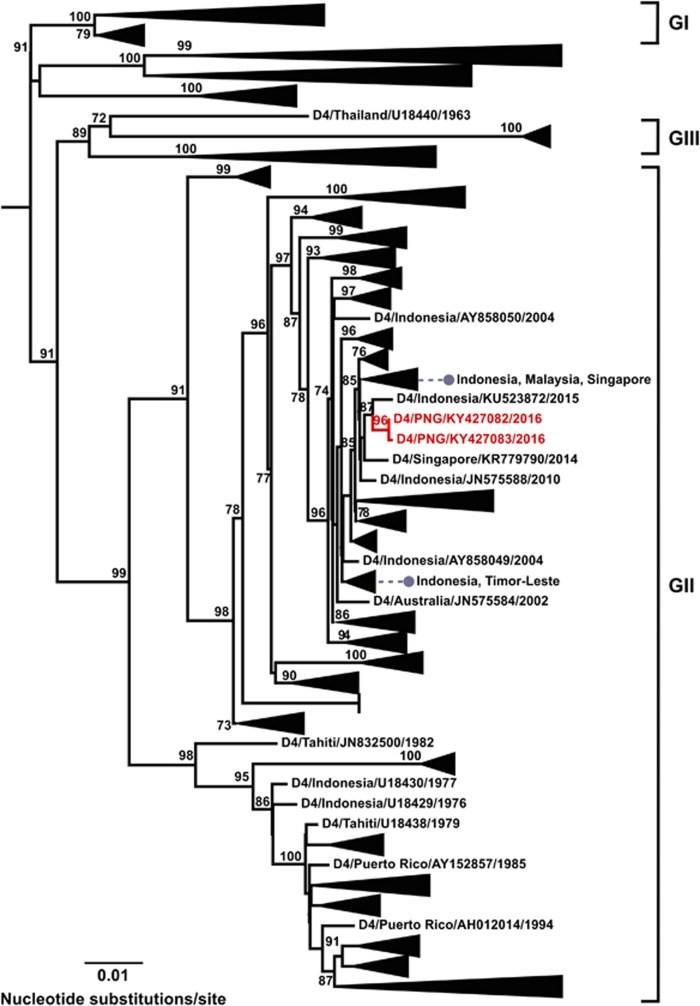
Maximum-likelihood phylogenetic tree of 1368 DENV-4 envelope (E) gene sequences (1485 nucleotides). Details of the phylogenetic tree analysis and graphical features are as given for Figure 2 legend. The phylogenetic tree includes two new PNG sequences in genotype II from viremic travelers who had returned to Australia in July and October, 2016. Genotype designations are as based on previously published groupings.^4, 5^

A comparatively smaller data set was available for DENV-4 and of the 1368 sequences we analyzed, 20 were new, including two from PNG. The two PNG DENV-4 sequences (GenBank accession numbers, KY427082 and KY427083) were sampled over 3 months apart in July and October 2016, respectively. They grouped together in genotype II and shared 99.9% nucleotide identity (Figure 5). These are the first known DENV-4 sequences reported from PNG and were most closely related to an Indonesian 2015 sequence (GenBank accession number KU523872).

### Contribution of PNG DENVs to dengue outbreaks in north Queensland, Australia

**Figure 6:**
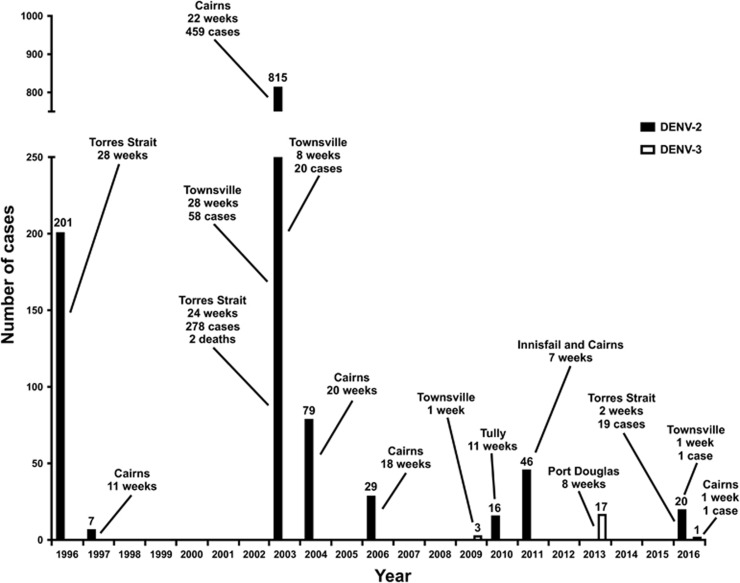
Dengue outbreaks in north Queensland, Australia, 1996–2016, caused by DENV-2 and DENV-3 importations from PNG. The geographic location, duration and number of cases are shown for each of the DENV-2 (solid black bars) and DENV-3 (outlined open bars) outbreaks. The Cairns 2016 outbreak involving a single case was caused by DENV-3.

**Figure 7:**
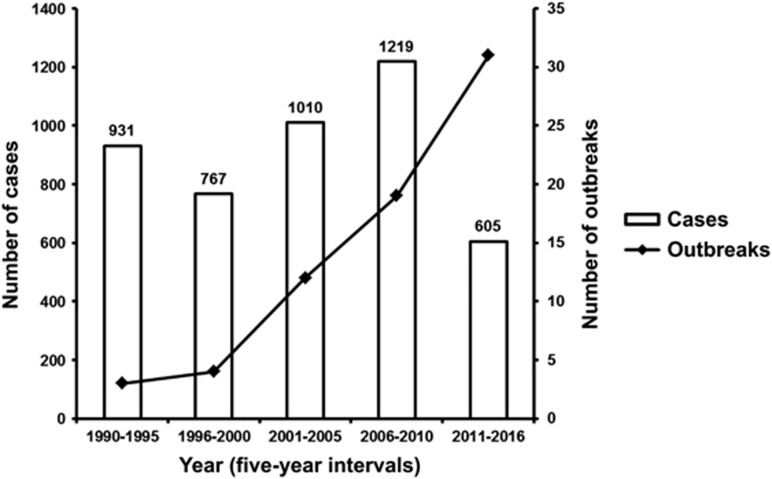
DENV 1–4 outbreaks occurring in north Queensland, Australia, 1990–2016. The total number of DENV 1–4 outbreaks and case numbers occurring in north Queensland, Australia between 1990 and 2016 are shown in 5-year intervals.

To ascertain the impact of DENV importations from PNG which have directly resulted in local outbreaks in north Queensland, Australia, we collated outbreak data for the period 1996–2016 including previously published information^29, 30, 33, 43^ (Figure 6). There were 12 geographically distinct outbreaks of DENV-2 and two outbreaks of DENV-3 recorded for the period. The year in which the largest number of cases occurred was 2003, which included four outbreaks and a total of 815 DENV-2 cases. The 2003 Torres Strait DENV-2 outbreak which lasted a total of 24 weeks, continued into early 2004 and resulted in two deaths associated with dengue hemorrhagic fever (DHF).^44^ The mean number of cases per outbreak for the first 10 years (1996–2005) was 157, while the mean number of cases per outbreak for the following 11 years (2006–2016) was 17. Although case numbers (*n*=132) were 89% lower in the period 2006–2016 relative to the number of cases in the period 1996–2005 (*n*=1102), the number of outbreaks occurring in the 2006–2016 period (*n*=8) was slightly higher. We next plotted this data with the total number of DENV 1–4 cases per outbreak from all countries, including PNG, occurring in north Queensland between 1990 and 2016 (in 5-year intervals, Figure 7). Since 2000, the number of outbreaks has increased. Paradoxically, reduced case numbers were reported for the period 2011–2016 compared to the preceding 20 years. Five of the outbreaks occurring between 2011 and 2016 (16%) were precipitated by DENV importations from PNG and three of these occurred in 2016 (Torres Strait and Townsville, DENV-2 and Cairns, DENV-3), albeit with relatively low-case numbers.

## DISCUSSION

Several factors constrain the current lack of an effective, functioning DENV surveillance system in PNG including geographically isolated communities, limited access to health services and inadequate mosquito control programs.^21^ Consequently, the true impact and disease burden caused by DENV in PNG is largely unknown. To date, only serological studies have provided evidence suggesting continual DENV transmission exists in PNG.^20, 45, 46^ The molecular data we present here are also limited by the periodical sampling of DENV strains imported into Australia from PNG. However, molecular assessment of circulating DENV strains is essential to understanding genetic diversity, transmission dynamics and disease risks within PNG. This knowledge is also important for assessing public health risks in regions like north Queensland, Australia who are susceptible to DENV outbreaks and regularly receive viremic travelers from PNG. Our data are therefore highly relevant for future dengue control initiatives and identifies specific endemic PNG DENV lineages which may have the potential for further dissemination throughout Oceania and Southeast Asian regions.

In this study, we performed a phylogenetic analysis of 196 new DENV 1–4 E gene sequences obtained from Australian patients, including 36 from travelers from PNG, over a 16 year period between 2001 and 2016. In comparison with other globally available DENV 1–4 sequences, our findings provide the first molecular evidence and characterization of co-circulating, endemic DENV 1–3 lineages in PNG and the first description of locally transmitted DENV-4 strains in PNG. Indeed, the co-circulation of multiple DENV serotypes and existence of endemicity within PNG is supported by several serological studies including recent evidence suggesting dengue has been a significant, yet neglected tropical disease in PNG for at least 70 years.^46^

Dengue disease outcomes during epidemics are confounded by the complex interplay that exists between immunological and transmission factors. The introduction of new DENV lineages displaying increased viral fitness and replication ability into dengue endemic regions has also been shown to correlate with heightened disease severity.^47, 48^ Surprisingly, severe disease due to DHF and dengue shock syndrome (DSS) has not been reported from PNG. Indeed, the paucity of severe dengue reports in PNG may reflect the difficulties in distinguishing between clinically similar manifestations, limited diagnostic support to identify etiological agents and/or underreporting of cases. However, two deaths attributable to DHF occurred in the Torres Strait, Australia, in 2004, following an outbreak of DENV-2 imported from PNG.^30, 44^ Hence our identification of newly introduced endemic lineages of multiple serotypes, together with the continual introduction of genetically diverse strains into PNG, highlights conditions that are conducive to increased disease severity and warrants improved monitoring and surveillance of DENV in PNG.

Considerable genetic diversity was apparent within the endemic PNG DENV 1–3 lineages we identified, as evidenced by multiple clades. With the exception of the DENV-1 PNG lineage, phylogenetic groupings within clades were largely characterized by a temporal pattern coinciding with the sequence sampling times. The DENV-3 endemic lineage suggested a clade replacement which may have occurred between 2010 and 2012. It is also likely that tree topologies were influenced by the geographical origin of where patients were infected and could have corresponded to variable geographic locations within PNG. Unfortunately, patient demographic data were unavailable for the current study, and we were unable to attempt further spatial correlation between sample location and the tree topologies observed for each of the endemic PNG DENV lineages. Nonetheless, spatial heterogeneity and migration have been shown to influence DENV dynamics in island regions.^49^

Due to its geographical proximity, we have previously identified PNG as being an important source of DENVs for Australia.^32^ Indeed, importations of DENVs from PNG into north Queensland led to 15 geographically separate outbreaks between 1996 and 2016. Our phylogenetic analysis has also identified potential spread of DENVs from PNG into other Pacific countries. Between 2000 and 2002, large outbreaks of DENV-1 occurred throughout the Pacific, due to multiple introductions of the virus from Asia, reaching Hawaii, Easter Island and Australia.^27, 50^ Although several Pacific Island nations reported outbreaks, no data were available from PNG. In this study, we show evidence that strains of DENV-1, which caused the Solomon Islands and Fiji outbreaks in 2002, and later on Yap Island in 2004, may have been circulating in PNG. In 2016, a large outbreak of DENV-2 was recorded in the Solomon Islands (http://reliefweb.int/disaster/ep-2016-000112-slb, Accessed 2 July 2017). The close relationship between the 2016 Solomon Islands DENV-2 strain (GenBank accession number KY495808) and an endemic PNG DENV-2 strain (GenBank accession number KT781551) suggests that PNG may have been the origin of this epidemic strain. Conversely, DENVs from elsewhere in the Pacific may have been introduced periodically into PNG as is evident from the phylogenetic relatedness of PNG DENV-3 strains (GenBank accession numbers KT758738, KT758776 and JN575571) with a recent Pacific DENV-3 clade, which is situated basally in relation to these three PNG DENV-3 strains and contained sequences sampled between 2012 and 2016 (Figure 4).

In addition to Australia and the Pacific, our analysis of globally available DENV 1–4 sequences suggests considerable intermixing of DENV 1–4 strains between PNG and Southeast Asia, in particular Indonesia. This reflects anthropogenic factors including increased air travel and shipping, as well as unchecked urbanization and lack of effective mosquito control programs. The global spread of *Ae. aegypti* and *Ae. albopictus* mosquito vectors has also extended to many Pacific regions and facilitated several DENV, CHIKV and ZIKV outbreaks.^23, 24, 28, 51, 52, 53, 54^ Concomitantly, several countries have experienced a dramatic increase in the frequency and magnitude of epidemic dengue in the last few decades.^55^ While case number trends have varied in north Queensland, Australia, the number of dengue outbreaks occurring between 2000 and 2016 has increased. This has largely been due to the increased importation of DENVs from viremic travelers, particularly from Bali and Southeast Asia.^32^

Ongoing surveillance and viral genetic studies are essential for tracking the emergence of pathogenic viruses, providing early warning of transmission and assessing potential development of endemicity. Our study is the first to identify established DENV lineages in PNG and characterize the molecular epidemiology of endemic PNG DENV strains. This new and definitive evidence of DENV endemicity in PNG has provided insights into the dynamics of DENV evolution and highlights PNG as a centre of DENV transmission in Australasia and the Western Pacific.

## Figures and Tables

**Table 1:** DENV 1–4 circulation in PNG based on sequence data, 2001–2016

**Serotype**	**Years**	**Endemic Lineage**^a^	**Additional PNG strains**^b^
DENV-1	2011–2016	Genotype IV KR919815/2011, KT824978/2011, KT825048/2011, KT824991/2012, KT825009/2013, KT825010/2013, KT825057/2013, KR919818/2014, KR919814/2014, KT825040/2015, KY495800/2015, KY495801/2016	JN415518/2003, Genotype IV JN415519/2009, Genotype I KT824977/2011, Genotype I KT824990/2012, Genotype I KT825008/2013, Genotype I
DENV-2	2009–2016	Cosmopolitan JN568241/2009, JN568267/2010, JN568268/2010/ JN568269/2010, KT781530/2011, KT806311/2011, KT781551/2013/ KT781553/2013, KT806320/2014, KT806326/2015, KY495811/2015, KY495812/2015, KY495810/2016, KY495813/2016	AY706002/2001, Cosmopolitan JN568266/2003, Cosmopolitan FJ906959/2008, Asian Genotype II JN568270/2010, Cosmopolitan KT806314/2013, Cosmopolitan KU517845/2013 Cosmopolitan
DENV-3	2010–2016	Genotype I JN575572/2010, JN575573/2010, KT758750/2012, KT758751/2012, KT758785/2013, KT758786/2013/ KT758760/2013, KT758775/2014, KT758797/2015, KY495824/2016	JN575571/2008, Genotype I KT758738/2011, Genotype I KT758749/2012, Genotype II KT758776/2014, Genotype I
DENV-4	2016		KY427082/2016, Genotype II KY427083/2016, Genotype II

a Genotypes, GenBank accession numbers and year of sequences belonging to established, endemic PNG lineages are shown for DENV 1–3.

b GenBank accession numbers, year and genotypes are given for additional sequences of circulating PNG strains.
