# Supplementary material for: Dengue viruses in Papua New Guinea: evidence of endemicity and phylogenetic variation, including the evolution of new genetic lineages
Source: Emerg Microbes Infect. 2017 Dec 20;6(12):e114–. doi: 10.1038/emi.2017.103 (PMC5750459; doi:10.1038/emi.2017.103)
Supplement: Supplementary Table S1 [file emi2017103x1.doc]

**Supplementary Table S1 Summary of new DENV 1-4 sequences obtained between 2001 and 2016 and PNG sequences used in the study**

| Serotype | Genotype | Year | GenBank accession number | Country/Territory | Strain |
| --- | --- | --- | --- | --- | --- |
| DENV-1 | I | 2009 | JN415519 | Papua New Guinea | PNG 2009 |
| DENV-1 | I | 2011 | KT824977* | Papua New Guinea | PNG 2011b |
| DENV-1 | I | 2011 | KT824980* | Sri Lanka | Sri Lanka 2011b |
| DENV-1 | I | 2011 | KT824981* | Thailand | Thailand 2011 |
| DENV-1 | I | 2012 | KT824985* | Cambodia | Cambodia 2012 |
| DENV-1 | I | 2012 | KT824990* | Papua New Guinea | PNG 2012a |
| DENV-1 | I | 2012 | KT824993* | Thailand | Thailand 2012a |
| DENV-1 | I | 2012 | KT824994* | Thailand | Thailand 2012b |
| DENV-1 | I | 2012 | KT824997* | Australia | Townsville 2012 |
| DENV-1 | I | 2012 | KT825050* | Australia | Cairns 2012 |
| DENV-1 | I | 2013 | KT825000* | Indonesia | Bali 2013c |
| DENV-1 | I | 2013 | KT825001* | Myanmar | Myanmar 2013 |
| DENV-1 | I | 2013 | KT825002* | Australia | Cairns 2013a |
| DENV-1 | I | 2013 | KT825006* | Australia | Innisfail 2013 |
| DENV-1 | I | 2013 | KT825008* | Papua New Guinea | PNG 2013a |
| DENV-1 | I | 2013 | KT825012* | French Polynesia | Tahiti 2013 |
| DENV-1 | I | 2013 | KT825017* | Vietnam | Vietnam 2013 |
| DENV-1 | I | 2014 | KT825028* | Sri Lanka | Sri Lanka 2014b |
| DENV-1 | I | 2014 | KT825031* | Thailand | Thailand 2014 |
| DENV-1 | I | 2014 | KT825059* | Indonesia | Bali 2014d |
| DENV-1 | I | 2014 | KT825061* | Indonesia | Bali 2014f |
| DENV-1 | I | 2015 | KY495792* | Thailand | Thailand 2015 |
| DENV-1 | I | 2015 | KT825035* | French Polynesia | French Polynesia 2015 |
| DENV-1 | I | 2015 | KT825038* | Malaysia | Malaysia 2015b |
| DENV-1 | I | 2015 | KT825044* | Vietnam | Vietnam 2015 |
| DENV-1 | I | 2016 | KY495793* | Vietnam | Vietnam 2016 |
| DENV-1 | IV | 2003 | JN415518 | Papua New Guinea | PNG 2003 |
| DENV-1 | IV | 2007 | KT824964* | Cook Islands | Cook Islands 2007 |
| DENV-1 | IV | 2011 | KR919815 | Papua New Guinea | PNG 2011 |
| DENV-1 | IV | 2011 | KT824976* | Australia | Innisfail 2011 |
| DENV-1 | IV | 2011 | KT824978* | Papua New Guinea | PNG 2011c |
| DENV-1 | IV | 2011 | KT824982* | Australia | Townsville 2011 |
| DENV-1 | IV | 2011 | KT825047* | Philippines | Philippines 2011 |
| DENV-1 | IV | 2011 | KT825048* | Papua New Guinea | PNG 2011d |
| DENV-1 | IV | 2012 | KY495797* | Fiji | Fiji 2012e |
| DENV-1 | IV | 2012 | KY495798* | Kiribati | Kiribati 2012b |
| DENV-1 | IV | 2012 | KY495799* | Niue Island | Niue Island 2012 |
| DENV-1 | IV | 2012 | KT824989* | Philippines | Philippines 2012b |
| DENV-1 | IV | 2012 | KT824991* | Papua New Guinea | PNG 2012b |
| DENV-1 | IV | 2013 | KT825007* | Philippines | Philippines 2013 |
| DENV-1 | IV | 2013 | KT825009* | Papua New Guinea | PNG 2013b |
| DENV-1 | IV | 2013 | KT825010* | Papua New Guinea | PNG 2013c |
| DENV-1 | IV | 2013 | KT825011* | Australia | Port Douglas 2013 |
| DENV-1 | IV | 2013 | KT825019* | Fiji | Fiji 2013 |
| DENV-1 | IV | 2013 | KT825057* | Papua New Guinea | PNG 2013d |
| DENV-1 | IV | 2014 | KR919814 | Papua New Guinea | PNG 2014a |
| DENV-1 | IV | 2014 | KR919818 | Papua New Guinea | PNG 2014b |
| DENV-1 | IV | 2015 | KY495800* | Papua New Guinea | PNG 2015c |
| DENV-1 | IV | 2015 | KT825036* | Australia | Innisfail 2015 |
| DENV-1 | IV | 2015 | KT825040* | Papua New Guinea | PNG 2015a |
| DENV-1 | IV | 2015 | KT825043* | Australia | Townsville 2015a |
| DENV-1 | IV | 2016 | KY495796* | Philippines | Philippines 2016 |
| DENV-1 | IV | 2016 | KY495801* | Papua New Guinea | PNG 2016 |
| DENV-1 | V | 2010 | KT824969* | India | India 2010b |
| DENV-1 | V | 2011 | KT824975* | India | India 2011 |
| DENV-1 | V | 2013 | KT825054* | Barbados | Barbados 2013 |
| DENV-1 | V | 2013 | KT825056* | Indonesia | Indonesia 2013b |
| DENV-1 | V | 2014 | KY495794* | Maldives | Maldives 2014b |
| DENV-1 | V | 2014 | KT825063* | Australia | Cairns 2014c |
| DENV-1 | V | 2014 | KT825065* | Singapore | Singapore 2014 |
| DENV-1 | V | 2015 | KT825041* | South America | Sth America 2015 |
| DENV-1 | V | 2015 | KT825042* | Thailand | Thailand 2015a |
| DENV-1 | V | 2015 | KT825067* | Australia | Cairns 2015 |
| DENV-1 | V | 2015 | KT825068* | India | India 2015 |
| DENV-1 | V | 2016 | KY495795* | Maldives | Maldives 2016 |
| DENV-2 | Asian I | 2013 | KT781555* | Australia | Mt Isa 2013 |
| DENV-2 | Asian I | 2013 | KT781556* | Thailand | Thailand 2013 |
| DENV-2 | Asian I | 2014 | KT806324* | Myanmar | Myanmar 2014 |
| DENV-2 | Asian I | 2015 | KY495819* | Cambodia | Cambodia 2015 |
| DENV-2 | Asian II | 1944 | AF038403 | Papua New Guinea | New Guinea C |
| DENV-2 | Asian II | 2008 | FJ906959 | Papua New Guinea | DENV-2/PG/BID-V2618/2008 |
| DENV-2 | Cosmopolitan | 2001 | AY706002 | Papua New Guinea | 7726 |
| DENV-2 | Cosmopolitan | 2003 | JN568266 | Papua New Guinea | PNG 2003 |
| DENV-2 | Cosmopolitan | 2003 | KY495815* | East Timor | East Timor 2003 |
| DENV-2 | Cosmopolitan | 2009 | JN568241 | Papua New Guinea | PNG 2009 |
| DENV-2 | Cosmopolitan | 2010 | JN568267 | Papua New Guinea | PNG 2010a |
| DENV-2 | Cosmopolitan | 2010 | JN568268 | Papua New Guinea | PNG 2010b |
| DENV-2 | Cosmopolitan | 2010 | JN568269 | Papua New Guinea | PNG 2010c |
| DENV-2 | Cosmopolitan | 2010 | JN568270 | Papua New Guinea | PNG 2010d |
| DENV-2 | Cosmopolitan | 2010 | KT781521* | Australia | Townsville 2010c |
| DENV-2 | Cosmopolitan | 2011 | KT781524* | Indonesia | Bali 2011a |
| DENV-2 | Cosmopolitan | 2011 | KT781527* | East Timor | East Timor 2011 |
| DENV-2 | Cosmopolitan | 2011 | KT781528* | Singapore | Singapore 2011 |
| DENV-2 | Cosmopolitan | 2011 | KT781530* | Papua New Guinea | PNG 2011a |
| DENV-2 | Cosmopolitan | 2011 | KT781532* | Bangladesh | Bangladesh 2011 |
| DENV-2 | Cosmopolitan | 2011 | KT781533* | Pakistan | Pakistan 2011 |
| DENV-2 | Cosmopolitan | 2011 | KT806311* | Papua New Guinea | PNG 2011c |
| DENV-2 | Cosmopolitan | 2011 | KT806312* | India | India 2011 |
| DENV-2 | Cosmopolitan | 2012 | KT781534* | Indonesia | Indonesia 2012 |
| DENV-2 | Cosmopolitan | 2012 | KT781537* | Thailand | Thailand 2012a |
| DENV-2 | Cosmopolitan | 2012 | KT781539* | Indonesia | Bali 2012c |
| DENV-2 | Cosmopolitan | 2012 | KT781541* | Australia | Townsville 2012 |
| DENV-2 | Cosmopolitan | 2013 | KT781544* | Indonesia | Indonesia 2013a |
| DENV-2 | Cosmopolitan | 2013 | KT781547* | Indonesia | Bali 2013c |
| DENV-2 | Cosmopolitan | 2013 | KT781548* | Indonesia | Bali 2013d |
| DENV-2 | Cosmopolitan | 2013 | KT781549* | Australia | Cairns 2013 |
| DENV-2 | Cosmopolitan | 2013 | KT781550* | Indonesia | Indonesia 2013b |
| DENV-2 | Cosmopolitan | 2013 | KT781551* | Papua New Guinea | PNG 2013a |
| DENV-2 | Cosmopolitan | 2013 | KT781553* | Papua New Guinea | PNG 2013c |
| DENV-2 | Cosmopolitan | 2013 | KT781554* | Indonesia | Indonesia 2013c |
| DENV-2 | Cosmopolitan | 2013 | KT806313* | Malaysia | Malaysia 2013 |
| DENV-2 | Cosmopolitan | 2013 | KT806314* | Papua New Guinea | PNG 2013d |
| DENV-2 | Cosmopolitan | 2013 | KU517845 | Papua New Guinea | PG-CN10-13 |
| DENV-2 | Cosmopolitan | 2014 | KT781557* | Indonesia | Indonesia 2014 |
| DENV-2 | Cosmopolitan | 2014 | KT781563* | Fiji | Fiji 2014a |
| DENV-2 | Cosmopolitan | 2014 | KT781564* | Philippines | Philippines 2014a |
| DENV-2 | Cosmopolitan | 2014 | KT781565* | India | India 2014 |
| DENV-2 | Cosmopolitan | 2014 | KT781567* | Vietnam | Vietnam 2014 |
| DENV-2 | Cosmopolitan | 2014 | KT806316* | Indonesia | Bali 2014b |
| DENV-2 | Cosmopolitan | 2014 | KT806317* | Thailand | Thailand 2014b |
| DENV-2 | Cosmopolitan | 2014 | KT806320* | Papua New Guinea | PNG 2014 |
| DENV-2 | Cosmopolitan | 2014 | KT806321* | Malaysia | Malaysia 2014c |
| DENV-2 | Cosmopolitan | 2014 | KT806323* | Malaysia | Malaysia 2014d |
| DENV-2 | Cosmopolitan | 2015 | KT781568* | Indonesia | Bali 2015 |
| DENV-2 | Cosmopolitan | 2015 | KT781569* | Singapore | Singapore 2015 |
| DENV-2 | Cosmopolitan | 2015 | KT781570* | East Timor | East Timor 2015 |
| DENV-2 | Cosmopolitan | 2015 | KT781571* | Philippines | Philippines 2015 |
| DENV-2 | Cosmopolitan | 2015 | KT781572* | Thailand | Thailand 2015 |
| DENV-2 | Cosmopolitan | 2015 | KT806325* | Malaysia | Malaysia 2015 |
| DENV-2 | Cosmopolitan | 2015 | KT806326* | Papua New Guinea | PNG 2015 |
| DENV-2 | Cosmopolitan | 2015 | KY495811* | Papua New Guinea | PNG 2015b |
| DENV-2 | Cosmopolitan | 2015 | KY495812* | Papua New Guinea | PNG 2015c |
| DENV-2 | Cosmopolitan | 2015 | KY495816* | East Timor | East Timor 2015b |
| DENV-2 | Cosmopolitan | 2016 | KY495802* | India | India 2016 |
| DENV-2 | Cosmopolitan | 2016 | KY495803* | Sri Lanka | Sri Lanka 2016 |
| DENV-2 | Cosmopolitan | 2016 | KY495804* | Australia | Cairns 2016 |
| DENV-2 | Cosmopolitan | 2016 | KY495805* | Thailand | Thailand 2016 |
| DENV-2 | Cosmopolitan | 2016 | KY495806* | Indonesia | Bali 2016 |
| DENV-2 | Cosmopolitan | 2016 | KY495807* | Australia | Charters Towers 2016 |
| DENV-2 | Cosmopolitan | 2016 | KY495808* | Solomon Islands | Solomon Islands 2016 |
| DENV-2 | Cosmopolitan | 2016 | KY495809* | Australia | Badu Island 2016 |
| DENV-2 | Cosmopolitan | 2016 | KY495810* | Papua New Guinea | PNG 2016a |
| DENV-2 | Cosmopolitan | 2016 | KY495813* | Papua New Guinea | PNG 2016b |
| DENV-2 | Cosmopolitan | 2016 | KY495814* | Australia | Townsville 2016 |
| DENV-3 | I | 2008 | JN575571 | Papua New Guinea | PNG 2008 |
| DENV-3 | I | 2009 | KY495825* | Australia | Cairns 2009 |
| DENV-3 | I | 2010 | JN575572 | Papua New Guinea | PNG 2010a |
| DENV-3 | I | 2010 | JN575573 | Papua New Guinea | PNG 2010b |
| DENV-3 | I | 2011 | KT758738* | Papua New Guinea | PNG 2011 |
| DENV-3 | I | 2011 | KT758739* | Philippines | Philippines 2011 |
| DENV-3 | I | 2012 | KT758743* | Australia | Cairns 2012 |
| DENV-3 | I | 2012 | KT758744* | Indonesia | Indonesia 2012 |
| DENV-3 | I | 2012 | KT758746* | East Timor | East Timor 2012b |
| DENV-3 | I | 2012 | KT758747* | Philippines | Philippines 2012 |
| DENV-3 | I | 2012 | KT758750* | Papua New Guinea | PNG 2012b |
| DENV-3 | I | 2012 | KT758751* | Papua New Guinea | PNG 2012c |
| DENV-3 | I | 2012 | KT758765* | Solomon Islands | Solomon Islands 2012 |
| DENV-3 | I | 2013 | KT758786* | Papua New Guinea | PNG 2013c |
| DENV-3 | I | 2013 | KT758754* | Indonesia | Bali 2013a |
| DENV-3 | I | 2013 | KT758756* | Indonesia | Bali 2013c |
| DENV-3 | I | 2013 | KT758757* | Indonesia | Indonesia 2013 |
| DENV-3 | I | 2013 | KT758758* | Indonesia | Bali 2013d |
| DENV-3 | I | 2013 | KT758760* | Papua New Guinea | PNG 2013a |
| DENV-3 | I | 2013 | KT758761* | Fiji | Fiji 2013 |
| DENV-3 | I | 2013 | KT758762* | Solomon Islands | Solomon Islands 2013a |
| DENV-3 | I | 2013 | KT758763* | Solomon Islands | Solomon Islands 2013b |
| DENV-3 | I | 2013 | KT758764* | Solomon Islands | Solomon Islands 2013c |
| DENV-3 | I | 2013 | KT758766* | Philippines | Philippines 2013 |
| DENV-3 | I | 2013 | KT758785* | Papua New Guinea | PNG 2013b |
| DENV-3 | I | 2013 | KT758788* | Australia | Port Douglas 2013b |
| DENV-3 | I | 2014 | KT758773* | Indonesia | Bali 2014 |
| DENV-3 | I | 2014 | KT758774* | Malaysia | Malaysia 2014 |
| DENV-3 | I | 2014 | KT758775* | Papua New Guinea | PNG 2014a |
| DENV-3 | I | 2014 | KT758776* | Papua New Guinea | PNG 2014b |
| DENV-3 | I | 2014 | KT758777* | Republic of Nauru | Nauru Island 2014 |
| DENV-3 | I | 2014 | KT758780* | Vanuatu | Vanuatu 2014b |
| DENV-3 | I | 2014 | KT758781* | Philippines | Philippines 2014 |
| DENV-3 | I | 2014 | KT758792* | Fiji | Fiji 2014 |
| DENV-3 | I | 2014 | KT758798* | Vanuatu | Vanuatu 2014c |
| DENV-3 | I | 2015 | KY499643* | Indonesia | Indonesia 2015 |
| DENV-3 | I | 2015 | KT758793* | Indonesia | Bali 2015a |
| DENV-3 | I | 2015 | KT758782* | Kingdom of Tonga | Tonga 2015a |
| DENV-3 | I | 2015 | KT758783* | Kingdom of Tonga | Tonga 2015b |
| DENV-3 | I | 2015 | KT758794* | Indonesia | Bali 2015b |
| DENV-3 | I | 2015 | KT758795* | Samoa | Samoa 2015 |
| DENV-3 | I | 2015 | KT758797* | Papua New Guinea | PNG 2015 |
| DENV-3 | I | 2015 | KT825075* | Philippines | Philippines 2015 |
| DENV-3 | I | 2016 | KY495820* | Malaysia | Malaysia 2016 |
| DENV-3 | I | 2016 | KT758796* | Solomon Islands | Solomon Islands 2016 |
| DENV-3 | I | 2016 | KY495822* | Philippines | Philippines 2016 |
| DENV-3 | I | 2016 | KY495824* | Papua New Guinea | PNG 2016 |
| DENV-3 | II | 2011 | KT758741* | Thailand | Thailand 2011b |
| DENV-3 | II | 2011 | KT758742* | Thailand | Thailand 2011c |
| DENV-3 | II | 2012 | KC261634 | China | GZ/10476/2012 |
| DENV-3 | II | 2012 | KT758748* | Thailand | Thailand 2012 |
| DENV-3 | II | 2013 | KT758790* | Thailand | Thailand 2013c |
| DENV-3 | III | 2012 | KT758749* | Papua New Guinea | PNG2012a |
| DENV-3 | III | 2012 | KT758752* | India | India 2012 |
| DENV-3 | III | 2013 | KT758768* | Indonesia | Bali 2013e |
| DENV-3 | III | 2013 | KT758769* | India | India 2013 |
| DENV-3 | III | 2013 | KT758770* | Cambodia | Cambodia 2013 |
| DENV-3 | III | 2013 | KT758771* | Thailand | Thailand 2013b |
| DENV-3 | III | 2013 | KT758772* | Singapore | Singapore 2013 |
| DENV-3 | III | 2015 | KY495823* | Thailand | Thailand 2015b |
| DENV-3 | III | 2015 | KT758784* | Thailand | Thailand 2015 |
| DENV-4 | I | 2011 | KT749998* | Thailand | Bangkok 2011 |
| DENV-4 | I | 2012 | KT750001* | Philippines | Philippines 2012b |
| DENV-4 | I | 2013 | KT750007* | Thailand | Thailand 2013 |
| DENV-4 | I | 2015 | KT825074* | Thailand | Thailand 2015 |
| DENV-4 | I | 2016 | KY427080* | Sri Lanka | Sri Lanka 2016 |
| DENV-4 | I | 2016 | KY427081* | Indonesia | Bali 2016b |
| DENV-4 | II | 2008 | KT749993* | Samoa | Samoa 2008c |
| DENV-4 | II | 2011 | KT749994* | Thailand | Thailand 2011 |
| DENV-4 | II | 2011 | KT749995* | Australia | Cairns 2011 |
| DENV-4 | II | 2011 | KT749997* | Philippines | Philippines 2011 |
| DENV-4 | II | 2012 | KY427078* | Federated States of Micronesia (Chuuk Island) | ChuukIsl 2012 |
| DENV-4 | II | 2012 | KT749999* | Philippines | Philippines 2012a |
| DENV-4 | II | 2012 | KT750000* | Federated States of Micronesia (Kosrae Island) | Kosrae 2012 |
| DENV-4 | II | 2013 | KT750003* | Indonesia | Bali 2013 |
| DENV-4 | II | 2013 | KT750004* | South America | Sth America 2013 |
| DENV-4 | II | 2013 | KT750005* | Philippines | Philippines 2013a |
| DENV-4 | II | 2016 | KY427077* | Indonesia | Bali 2016a |
| DENV-4 | II | 2016 | KY427079* | Australia | Cairns 2016 |
| DENV-4 | II | 2016 | KY427082* | Papua New Guinea | PNG 2016a |
| DENV-4 | II | 2016 | KY427083* | Papua New Guinea | PNG 2016b |

*New DENV sequences obtained from viremic travelers who traveled to Australia or from patients diagnosed during local DENV transmission events between 2001 and 2016.
